# Efficacy and safety of ciprofol for sedation in outpatient gynecological procedures: a phase III multicenter randomized trial

**DOI:** 10.3389/fmed.2024.1360508

**Published:** 2024-04-23

**Authors:** Jing Xu, Mengchang Yang, Yuan Zeng, Xiao-Hua Zou, Jing-Hua Ren, Zhongyuan Xia, Hai-Hui Xie, Yong-Hao Yu, Ming-Jun Xu, Wei Chen, Dong-Xin Wang

**Affiliations:** ^1^Department of Anesthesiology, Peking University First Hospital, Beijing, China; ^2^Department of Anesthesiology, Sichuan Provincial People’s Hospital, Chengdu, China; ^3^Department of Anesthesiology, Affiliated Hospital of Guizhou Medical University, Guiyang, China; ^4^Department of Anesthesiology, Yibin Second People’s Hospital, Yibin, China; ^5^Department of Anesthesiology, Renmin Hospital of Wuhan University, Wuhan, China; ^6^Department of Anesthesiology, Dongguan People’s Hospital, Dongguan, China; ^7^Department of Anesthesiology, Tianjin Medical University General Hospital, Tianjin, China; ^8^Department of Anesthesiology, Beijing Obstetrics and Gynecology Hospital, Beijing, China; ^9^Haisco Pharmaceutical Group Co., Ltd., Chengdu, China; ^10^Outcomes Research Consortium, Cleveland, OH, United States

**Keywords:** ciprofol, propofol, sedation, outpatient, gynecological procedure

## Abstract

**Objective:**

Ciprofol (also known as cipepofol and HSK3486), is a compound similar to propofol in chemical structure and hypnotic effect. Herein we evaluated the efficacy and safety of ciprofol for sedation in outpatient gynecological procedures.

**Methods:**

This phase III multicenter randomized trial with a non-inferiority design was conducted in nine tertiary hospitals. We enrolled 135 women aged 18–65 years who were scheduled for ambulatory gynecological procedures. Patients were randomly assigned to receive either ciprofol (0.4 mg/kg for induction and 0.2 mg/kg for maintenance) or propofol (2.0 mg/kg for induction and 1.0 mg/kg for maintenance) sedation in a 2:1 ratio. Patients and investigators for data collection and outcome assessment were blinded to study group assignments. The primary outcome was the success rate of sedation, defined as completion of procedure without remedial anesthetics. The non-inferiority margin was set at −8%. Secondary outcomes included time to successful induction, time to full awake, time to meet discharge criteria, and satisfaction with sedation assessed by patients and doctors. We also monitored occurrence of adverse events and injection pain.

**Results:**

A total of 135 patients were enrolled; 134 patients (90 patients received ciprofol sedation and 44 patients propofol sedation) were included in final intention-to-treat analysis. The success rates were both 100% in the two groups (rate difference, 0.0%; 95% CI, −4.1 to 8.0%), i.e., ciprofol was non-inferior to propofol. When compared with propofol sedation, patients given ciprofol required more time to reach successful induction (median difference [MD], 2 s; 95% CI, 1 to 7; *p* < 0.001), and required more time to reach full awake (MD, 2.3 min; 95% CI, 1.4 to 3.1; *p* < 0.001) and discharge criteria (MD, 2.3 min; 95% CI, 1.5 to 3.2; *p* < 0.001). Fewer patients in the ciprofol group were dissatisfied with sedation (relative risk, 0.21; 95% CI, 0.06 to 0.77; *p* = 0.024). Patients given ciprofol sedation had lower incidences of treat-emergent adverse events (34.4% [31/90] vs. 79.5% [35/44]; *p* < 0.001) and injection pain (6.7% [6/90] vs. 61.4% [27/44]; *p* < 0.001).

**Conclusion:**

Ciprofol for sedation in ambulatory gynecological procedures was non-inferior to propofol, with less adverse events and injection pain.

**Clinical trial registration:**

ClinicalTrials.gov, identifier NCT04958746.

## Introduction

Propofol, chemically named 2, 6-diisopropyl phenol, produces hypnotic effect mainly by enhancing gamma-aminobutyric acid type A (GABA_A_) receptor-mediated inhibitory synaptic currents. Although propofol is broadly used for sedation and general anesthesia, the related adverse effects including injection pain, respiratory suppression, and circulatory inhibition cannot be ignored. Compounds chemically like propofol were produced after incorporating cyclopropyl group into the 2,6-side chain to increase its lipophilicity, and introducing one or more chiral centers to break the structure symmetry. The aim was to reduce propofol-related side effects while retaining sedative efficacy. Among these compounds, ciprofol (also known as cipepofol and HSK3486) was identified as a novel anesthetic agent (1). Like propofol, ciprofol exerts hypnotic effects by acting on GABA_A_ receptors and produces rapid-onset action and clear wake-up with similar pharmacokinetic characteristics of absorption, distribution, metabolism (2).

Ciprofol has been evaluated in clinical trials from phase I (3, 4), phase II (5–7), to phase III (8–10). In a phase I trial, ciprofol 0.4–0.9 mg/kg produced rapid onset of anesthesia and rapid recovery of consciousness, and was well tolerated by subjects (4). In a phase II trial, the efficacy and safety of ciprofol 0.4–0.5 mg/kg were comparable to propofol 2 mg/kg in adults under 65 years of age (6). In phase III trials, the success rates of ciprofol were all 100% when used for sedation in digestive endoscopy (6, 8) and fiberoptic bronchoscopy (9), for anesthesia induction in elective gynecological surgery (11), and for anesthesia maintenance in elective surgery (12, 13). Furthermore, ciprofol induction produced a more stable change of bispectral index and slighter variations of blood pressure and heart rate (10). The indications for *sedation in digestive endoscopy* (approval number H20200013) and *general anesthesia* (approval number H20210007) have been approved by the National Medical Products Administration of China.^1^ Up to now, ciprofol has been used for anesthesia in aged patients (14), patients with mild to moderate liver injury (15), and patients undergoing kidney transplantation (16); it has also been used for sedation in intensive care unit patients with mechanical ventilation (7).

As a novel anesthetic agent, the efficacy and safety of ciprofol requires further demonstration. Outpatient gynecological procedures usually last for 2 to 30 min under sedation. The use of ciprofol for sedation in outpatients undergoing gynecological procedure has not been evaluated. We therefore designed this non-inferiority trial to compare the efficacy and safety of ciprofol versus propofol in gynecological outpatients.

## Materials and methods

This phase III multicenter randomized parallel-group trial was conducted in nine tertiary hospitals across China. The trial protocol was approved by the Biomedical Research Ethics Committee of Peking University First Hospital (2021-052, June 25, 2021) and other participating centers and was prospectively registered with ClinicalTrials.gov (NCT04958746; June 23, 2021). Written informed consent was obtained from each participating patient.

### Participants

We included patients aged between 18–65 years who had a body mass index (BMI) between 18–30 kg/m^2^ and an American Society of Anesthesiologists (ASA) classification I to II and were scheduled for gynecological procedures under intravenous sedation. We excluded patients who had contraindications to general anesthesia or a history of previous anesthesia incidents; were allergic to propofol injection, ciprofol injection, excipients of study drugs, and opioids or their drug ingredients; had positive urine or blood human chorionic gonadotropin (HCG) test (except abortion, curettage, or other outpatient procedures for pregnancy termination); or were in lactating period. Detailed inclusion and exclusion criteria are listed in Supplementary File S1.

### Randomization, masking, and study drug administration

Random allocations were generated in a 2:1 ratio with a block size of nine by an independent statistician using the SAS 9.4 software (SAS Institute, United States). During the study period, random numbers were obtained from the Rave Data Management/Randomization and Trial Supply Management (RTSM; MediData Institute, United States) by non-blinded pharmacists who prepared the study drugs but were otherwise not involved in the trial. The study drugs, either ciprofol (0.25% ciprofol injection, 20 mL/ampoule; Haisco, Xingcheng, Liaoning, China) or propofol (1% propofol injection, 20 mL/ampoule; AstraZeneca, Wuxi, Jiangsu, China), were provided as emulsion for injection with identical appearance to responsible anesthesiologists. In this way the enrolled patients were randomly assigned to receive either ciprofol or propofol anesthesia in a 2:1 ratio. For each participant, a non-blinded anesthesiologist was designated for study drug administration. All patients, other health-care team members, and investigators who were responsible for data collection and outcomes assessment were blinded to study group assignments.

Intraoperative monitoring included electrocardiogram, noninvasive blood pressure, respiratory rate, and pulse oxygen saturation (SpO_2_). Anesthesia was induced after mask oxygen inhalation at 5 L/min for 3 min. Fentanyl 50 μg was injected over 10 ± 5 s. Ciprofol 0.4 mg/kg or propofol 2.0 mg/kg was then injected over 30 ± 5 s. The initiation of study drug administration was marked as 0 min and followed by evaluation with Modified Observer’s Assessment of Alert/Sedation (MOAA/S; Supplementary Table S1) every 30 ± 10 s. If the required depth of sedation (MOAA/S score of ≤1) was not reached in 2 min, additional ciprofol 0.2 mg/kg or propofol 1.0 mg/kg was injected over 10 ± 5 s until the required depth was achieved. If adequate sedation was not achieved with the above study drug doses, propofol was added as a remedy. Gynecological procedures began after successful induction (MOAA/S ≤1). During the procedure, 5 L/min oxygen inhalation was given via anesthetic mask and patients were manually ventilated, when necessary, without the use of endotracheal tube or laryngeal mask.

During the procedures (started with speculum placement and ended with speculum removal), additional ciprofol 0.2 mg/kg or propofol 1.0 mg/kg was injected when there were clinical signs indicating light sedation (body movement or eye opening). In case that more than 5 additional doses of study drugs were required within any consecutive 15 min, propofol would be administered as a remedy. For each case, an independent anesthesiologist who was not involved in the trial monitored vital signs and assured safety. At the end of procedures, patients were monitored in the operating room until regain consciousness, and then in the post-anesthesia care unit (PACU) for at least 30 min until fully awake and Aldrete score ≥9. Patients were then discharged to home.

### Data collection and outcome assessment

Baseline data included demographic and morphometric characteristics, previous comorbidities, history of smoking and alcohol drinking, and results of relevant laboratory tests and other examinations. Intraoperative data included doses of study drugs and remedial medications, vital signs, verbal rating scale (VRS) (0-no pain; 1-mild pain; 2-moderate pain; 3-severe pain) for injection pain, and type and duration of procedure. Intraoperative vital signs, including mean arterial pressure (MAP), heart rate (HR), and pulse oxygen saturation (SpO_2_), were monitored every 2 min before induction, every minute during study drug administration until awaking, and every 2 min after awaking. Averages of the first three recorded values under resting state before induction were adopted as baseline vital sign values.

Our primary outcome was the success rate of sedation, defined as completion of procedure without remedial anesthetics. Secondary outcomes included (1) time to successful induction (defined as interval from the first study drug administration to the first MOAA/S score ≤1); (2) time to full awake (defined as interval from the last study drug administration to completely awaking, as indicated by the first MOAA/S = 5 in three consecutive MOAA/S = 5 assessments); (3) time to meet discharge criteria (defined as interval from the last study drug administration to the first Aldrete score ≥9 in three consecutive Aldrete score ≥9 assessments); (4) satisfaction with sedation as assessed by patients (Supplementary Table S2), anesthesiologists (Supplementary Table S3) and surgeons (Supplementary Table S4).

Safety outcomes including treatment-emergent adverse events (TEAEs) and treatment-related adverse events (TRAEs) were followed up and recorded from the screening period until 2–4 days after surgery. The severity of adverse events was classified into five grades, i.e., grade 1 (mild), grade 2 (moderate), grade 3 (severe), grade 4 (life-threatening) and grade 5 (causing death), according to the Common Terminology Criteria for Adverse Events (CTCAE version 5.0). For each individual patient, the most serious adverse event was recorded.

Predefined adverse events potentially related to study drug administration included respiratory depression (respiratory rate <8 breaths/min), desaturation (SpO_2_ <95% with oxygen inhalation), apnea (absence of breathing action for more than 10 s), tachypnea (respiratory rate >20 breaths/min), hypotension (systolic pressure <90 mmHg, diastolic pressure <50 mmHg, or MAP decrease of ≥20% from baseline), bradycardia (heart rate <50 beats/min or a decrease of >30% from baseline), tachycardia (heart rate >100 beats/min or an increase of >30% from baseline), and new-onset arrhythmia requiring therapy. We also recorded nausea, vomiting, dizziness, injection pain, postoperative pain, skin rash, and other adverse events that emerged after study drug administration. Injection pain was evaluated during administration of the first dose of study drugs with a 4-point scale (0 = no pain; 1 = mild pain; 2 = moderate pain; 3 = severe pain). Postoperative pain was evaluated after full awake with a 10-point verbal rating scale (0 = no pain and 10 = the worst pain).

### Statistical analysis

#### Sample size estimation

The success rate of propofol sedation was assumed to be 99% and the non-inferiority margin was set at −8%. A sample size of 135 patients was required to test the difference between two groups with 80% power at a one-sided significance level of 0.025, considering a dropout rate of 10%. We assigned patients to the ciprofol group (*N* = 90) or the propofol group (*N* = 45) in a ratio of 2:1. Sample size calculation was done with the SAS 9.4 software.

#### Data analysis

Outcome analyses were primarily performed in the intention-to treat population, that is all patients were analyzed in the group to which they were randomized. For the primary outcome, analysis was also performed in the per-protocol population, excluding patients who dropped out of the trial. Safety analyses were based on the safety population, including patients who had received at least one study drug administration.

Baseline balance was assessed using absolute standardized difference (ASD), that is the absolute differences in means, mean ranks, or proportions divided by the pooled standard deviation. Variables with an ASD >0.360 (calculated by $1.96\times \sqrt{\left(n_{1}+n_{2}\right)/\left(n_{1}\times n_{2}\right)}$ ; *n*_1_ and *n*_2_ were the number of patients in each randomized group) were considered imbalanced (17).

For the primary outcome, the difference of successful sedation rates between groups was presented as rate difference and 95% confidence interval (CI), and compared with the non-inferiority margin.

For secondary outcomes, discrete variables were analyzed with the Mann–Whitney *U* test; median difference (95% CI) was calculated with the Hodges–Lehmann estimator. Categorical variables were analyzed with chi-square or Fisher exact tests and the differences between groups were presented as relative risks and 95% CIs. Missing data were not replaced.

For all hypotheses, two-tailed *p*-values <0.05 were considered statistically significant. Statistical analyses were performed with the SPSS 25.0 (IBM SPSS Inc., United States) software package.

## Results

### Patients

**Figure 1:**
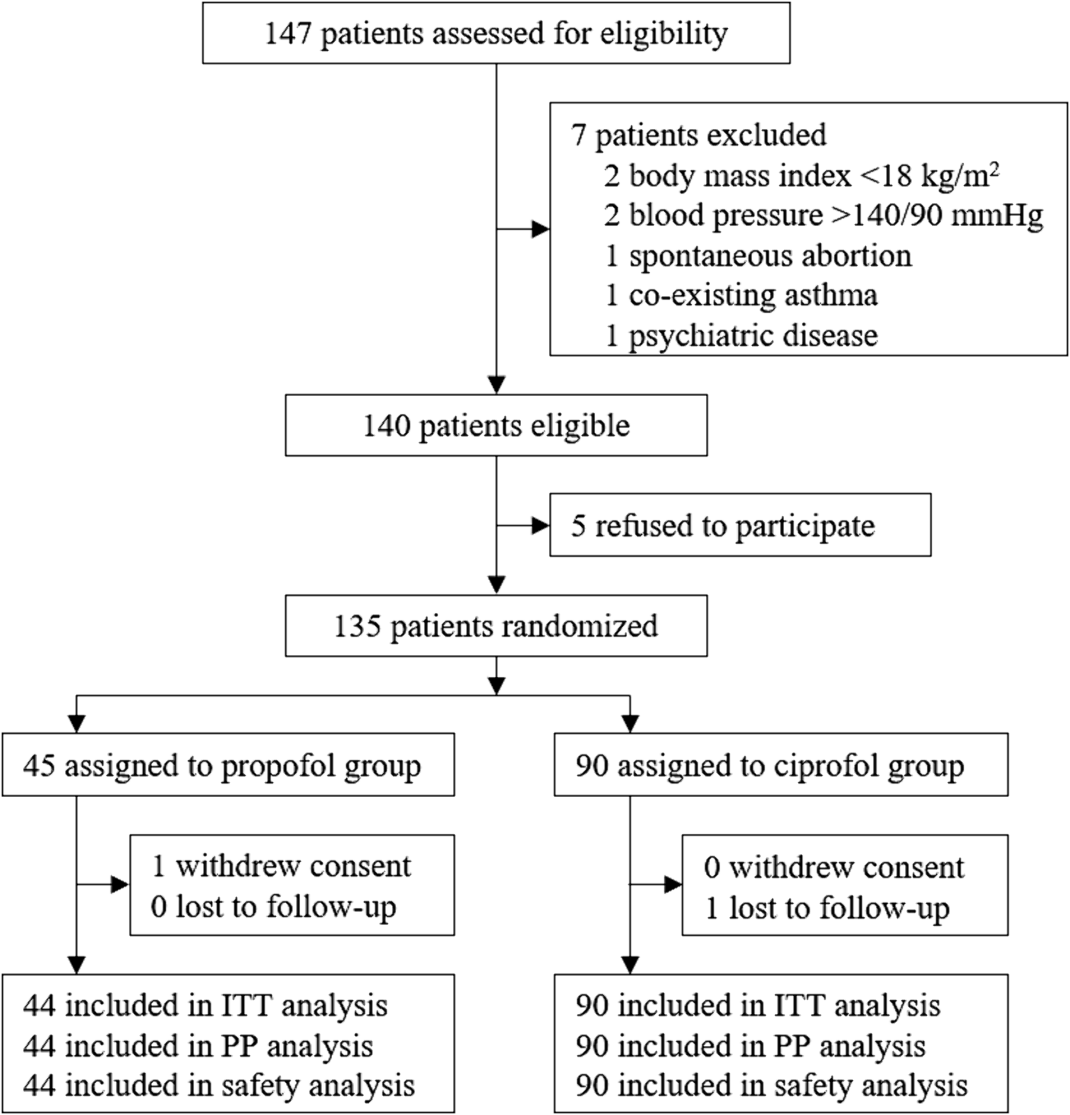
Trial profile.

From July 26, 2021 to September 30, 2021, a total of 147 patients were screened for eligibility. Among them, 140 patients met the inclusion/exclusion criteria; 135 patients were enrolled and randomly assigned to the propofol (*N* = 45) or the ciprofol group (*N* = 90). One patient in the propofol group withdrew consent before receiving study drug. Thus, 134 patients received at least one study drug dose and were included in the intention-to-treat, per-protocol, and safety analyses (Figure 1).

### Baseline and perioperative data

Baseline characteristics were generally balanced between the two groups, except that the proportions with histories of artificial abortion and with negative HCG-test results were higher in the ciprofol group (Table 1). Intraoperative data including the number of additional study drug dosages and the number of maximal study drug dosages within any 15 min were comparable between the two groups (Table 2).

**Table 1:** Baseline data.

	Propofol (*N* = 44)	Ciprofol (*N* = 90)	Absolute standardized difference
Age (year)	34.9 (9.4)	34.2 (9.1)	0.076
Body mass index (kg m^−2^)	22.1 (2.7)	21.9 (2.5)	0.077
*History of surgery*			
Artificial abortion	11 (25.0)	38 (42.2)	**0.370**
Caesarean section	15 (34.1)	29 (32.2)	0.040
Intrauterine device implantation	6 (13.6)	6 (6.7)	0.230
History of general anesthesia	18 (40.9)	43 (47.8)	0.139
History of allergy	4 (9.1)	15 (16.7)	0.228
History of diseases	34 (77.3)	81 (90.0)	0.349
Vaginal infection	15 (34.1)	23 (25.6)	0.187
Leiomyoma of uterus	6 (13.6)	12 (13.3)	0.009
Abnormal uterine bleeding	1 (2.3)	9 (10.0)	0.325
*Concomitant medications*			
Oxytocin	17 (38.6)	28 (31.1)	0.158
Cephalosporin antibiotic	20 (45.5)	29 (32.2)	0.275
Azithromycin	7 (15.9)	10 (11.1)	0.141
Ornidazole/metronidazole	11 (25.0)	19 (21.1)	0.093
Estrogen/progestin	10 (22.7)	28 (31.1)	0.190
Chinese traditional medicine	15 (34.1)	35 (38.9)	0.100
Fosfomycin trometamol	6 (13.6)	10 (11.1)	0.076
Test result of HCG			**0.361**
Negative	8 (18.2)	30 (33.3)	
Positive	32 (72.7)	56 (62.2)	
Not tested	4 (9.1)	4 (4.4)	
ASA classification			0.101
Class I	40 (90.9)	79 (87.8)	
Class II	4 (9.1)	11 (12.2)	
Modified Mallampati score			0.254
I	36 (81.8)	64 (71.1)	
II	8 (18.2)	26 (28.9)	

Data are mean (standard deviation) or *n* (%). HCG, human chorionic gonadotropin; ASA, American Society of Anesthesiologists. Absolute standardized differences in bold indicated >0.360 and were considered imbalanced between the two groups.

**Table 2:** Intraoperative data.

	Propofol (*N* = 44)	Ciprofol (*N* = 90)	*P*-value
First dose (mg)	2 [2, 2]	0.4 [0.4, 0.4]	—
Total dose (mg)	3 [2, 3]	0.6 [0.4, 0.8]	—
Additional dosages (count)	1 [1, 2]	1 [1, 2]	0.284
Frequency of additional dosages			0.496
0	16 (36.3)	30 (33.3)	
1	20 (45.5)	32 (35.6)	
2	5 (11.4)	17 (18.9)	
3	3 (6.8)	11 (12.2)	
Max dosages within any 15 min (count)	2 [1, 2]	2 [1, 3]	0.264
Remedial medication	0 (0)	0 (0)	—
Type of procedures			
Abortion	30 (68.2)	61 (67.8)	0.962
Cervical dilatation and uterine curettage	5 (11.3)	18 (20.0)	0.213
Implantation or removal of intrauterine device	17 (38.6)	21 (23.3)	0.065
Hysteroscopy	5 (11.4)	7 (7.8)	0.495
Others^a^	5 (11.4)	11 (12.2)	0.886
Duration of procedure (min)	5.0 [3.4, 7.9]	5.2 [3.4, 8.1]	0.903

Data are median [interquartile range] or *n* (%).

a Including cervical polypectomy, uterine polypectomy, and cervical biopsy.

### Efficacy outcomes

The success rate of sedation was 100% in both groups (rate difference, 0.0%; 95% CI, −4.1 to 8.0%; *p* > 0.999) (Table 3). With the lower limit of 95% CI greater than the preset non-inferiority margin −8%, the assumption that ciprofol was not inferior to propofol in anesthetic efficacy was manifested.

**Table 3:** Efficacy outcomes.

	Propofol (*N* = 44)	Ciprofol (*N* = 90)	Effect size (95% CI)^a^	*P*-value
**Primary outcome**				
Success rate of anesthesia^b^	44 (100.0)	90 (100.0)	Rate difference = 0.0 (−4.1 to 8.0)	>0.999
**Secondary outcomes**				
Time to successful induction (sec)^c^	60 [52, 60]	60 [60, 63]	Median difference = 2 (1 to 7)	**<0.001**
Time to full awake (min)^d^	7.7 [6.4, 9.1]	10.0 [8.5, 11.7]	Median difference = 2.3 (1.4 to 3.1)	**<0.001**
Time to PACU discharge (min)^e^	11.6 [10.4, 13.1]	14.1 [12.5, 15.7]	Median difference = 2.3 (1.5 to 3.2)	**<0.001**
**Satisfaction scores (score)** ^f^				
Patients	10 [10, 10] [6, 10]	10 [10, 10] [6, 10]	Median difference = 0 (0 to 0)	**0.011**
Anesthesiologists	12 [11, 12] [6, 12]	12 [11, 12] [4, 12]	Median difference = 0 (0 to 0)	0.872
Surgeons	12 [12, 12] [8, 12]	12 [12, 12] [5, 12]	Median difference = 0 (0 to 0)	0.286
**Dissatisfaction** ^g^				
Patients	7 (15.9)	3 (3.3)	Relative risk = 0.21 (0.06 to 0.77)	**0.024**
Anesthesiologists	13 (29.5)	23 (25.6)	Relative risk = 0.87 (0.49 to 1.54)	0.625
Surgeons	6 (13.6)	19 (21.1)	Relative risk = 1.55 (0.67 to 3.60)	0.297
**Exploratory outcomes**				
Mean arterial pressure (mmHg)^h^	79 (4)	83 (4)	Median difference = 4 (2 to 5)	**<0.001**
Heart rate (bpm)^h^	71 (3)	70 (3)	Median difference = −1 (−3 to 0)	0.149
Pulse oxygen saturation (%)^h^	97 [96, 97] [94, 98]	97 [96, 97] [95, 98]	Median difference = 0 (0 to 1)	0.097

Data are mean (standard deviation), median [interquartile range] [full range] or *n* (%). CI, confidence interval; PACU, post-anesthesia care unit. *P* values in bold indicate statistical differences between the two groups.

a Calculated as ciprofol group vs. or minus propofol group.

b Defined as the completement of surgery without anesthetic remedy.

c Defined as the duration from the first administration of the study drugs to the first Modified Observer’s Assessment of Alertness and Sedation (MOAA/S, a 6-point scale where 5 = alert and 0 = no responsiveness) score ≤1.

d Defined as the duration from the last administration of the study drugs to completely awaking, indicated by the first MOAA/S = 5 in three consecutive times of MOAA/S = 5 assessments.

e Defined as the duration from the last administration of the study drugs to departure, indicated by the first Aldrete score ≥9 in three consecutive times of Aldrete score assessments.

f Scores ranges from 0 (least satisfied) to 10 (fully satisfied) as for patients and ranges from 0 (least satisfied) to 12 (fully satisfied) as for anesthesiologists and surgeons.

g Satisfaction score <10 for patients and <12 for anesthesiologists and surgeons.

h Average value from the first administration of study drugs until awake from anesthesia.

Among secondary outcomes, time to successful induction was longer in patients receiving ciprofol (median 60 s (interquartile range 60 to 63) with ciprofol vs. 60 s [52 to 60] with propofol; median difference, 2 s; 95% CI, 1 to 7; *p* < 0.001). After procedure, patients in the ciprofol group required more time to reach full awake (10 min [8.5 to 11.7] with ciprofol vs. 7.7 min [6.4 to 9.1] with propofol; median difference, 2.3 min; 95% CI, 1.4 to 3.1; *p* < 0.001) and criteria for PACU discharge (14.1 min [12.5 to 15.7] with ciprofol vs. 11.6 min [10.4 to 13.1] with propofol; median difference, 2.3 min; 95% CI, 1.5 to 3.2; *p* < 0.001). Patients given ciprofol showed lower dissatisfaction rate (3.3% [3 of 90] with ciprofol vs. 15.9% [7 of 44] with propofol; relative risk, 0.21; 95% CI, 0.06 to 0.77; *p* = 0.024). There were no significant differences in satisfaction scores and dissatisfaction rates of both anesthesiologists and surgeons between the two groups (Table 3).

Among exploratory outcomes, patients receiving ciprofol had higher MAP during the procedure (83 ± 4 mmHg with ciprofol vs. 79 ± 4 mmHg with propofol; mean difference, 4 mmHg; 95% CI, 2 to 5; *p* < 0.001). There were no significant differences in HR and SpO_2_ during the procedure between the two groups (Table 3 and Supplementary Figures S1–S3).

### Safety outcomes

The incidence of TEAE (34.4% [31 of 90] with ciprofol vs. 79.5% [35 of 44] with propofol; relative risk, 0.46; 95% CI, 0.34 to 0.63; *p* < 0.001) and the severity of TEAE (median 0 [interquartile range 0, 1] with ciprofol vs. 2 [1, 2] with propofol; median difference, −1; 95% CI, −1 to −1; *p* < 0.001) were both lower in the ciprofol group than in the propofol group. Specifically, patients given ciprofol developed less desaturation (relative risk, 0.21; 95% CI, 0.06 to 0.77; *p* = 0.024) and any respiratory adverse events (relative risk, 0.29; 95% CI, 0.14 to 0.62; *p* < 0.001). The incidence of TRAE (27.8% [25 of 90] with ciprofol vs. 68.2% [30 of 44] with propofol; relative risk, 0.41; 95% CI, 0.28 to 0.60; *p* < 0.001) and the severity of TRAE (0 [0, 1] with ciprofol vs. 1 [1, 2] with propofol; median difference, −1; 95% CI, −1 to −1; *p* < 0.001) were also both lower in the ciprofol group. Patients in ciprofol group experienced less injection pain (6.7% [6 of 90] with ciprofol vs. 61.4% [27 of 44] with propofol; relative risk, 0.11; 95% CI, 0.05 to 0.24; *p* < 0.001) and lower severity of injection pain (0 [0, 0] with ciprofol vs. 1 [0, 2] with propofol; median difference, −1; 95% CI, −1 to −1; *p* < 0.001; Table 4).

**Table 4:** Safety outcomes.

	Propofol (*N* = 44)		Ciprofol (*N* = 90)		Effect size (95% CI)^a^	*P*-value
	Number of events^b^	Number of patients	Number of events^b^	Number of patients		
TEAE	55	35 (79.5)	48	31 (34.4)	Relative risk = 0.46 (0.34 to 0.63)	**<0.001**
Influenza like illness^c^	0	0 (0)	1	1 (1.1)	Relative risk = 0.99 (0.97 to 1.01)	>0.999
Respiratory depression^d^	5	4 (9.1)	10	6 (6.7)	Relative risk = 0.73 (0.22 to 2.47)	0.880
Desaturation^e^	8	7 (15.9)	3	3 (3.3)	Relative risk = 0.21 (0.06 to 0.77)	**0.024**
Apnea^f^	3	3 (6.8)	1	1 (1.1)	Relative risk = 0.16 (0.02 to 1.52)	0.200
Tachypnea^g^	1	1 (2.3)	0	0 (0)	Relative risk = 1.02 (0.98 to 1.07)	0.328
Hypotension^h^	4	3 (6.8)	7	5 (5.6)	Relative risk = 0.82 (0.20 to 3.26)	>0.999
Bradycardia^i^	1	1 (2.3)	4	3 (3.3)	Relative risk = 1.47 (0.16 to 13.70)	>0.999
Tachycardia^j^	0	0 (0)	1	1 (1.1)	Relative risk = 0.99 (0.97 to 1.01)	>0.999
Abnormal Q-wave^k^	0	0 (0)	1	1 (1.1)	Relative risk = 0.99 (0.97 to 1.01)	>0.999
Dizziness^c^	1	1 (2.3)	5	5 (5.6)	Relative risk = 2.44 (0.29 to 20.30)	0.663
Nausea^l^	0	0 (0)	1	1 (1.1)	Relative risk = 0.99 (0.97 to 1.01)	>0.999
Skin rash^m^	2	2 (4.5)	2	2 (2.2)	Relative risk = 0.49 (0.07 to 3.36)	0.840
Postoperative pain^n^	0	0 (0)	1	1 (1.1)	Relative risk = 0.99 (0.97 to 1.01)	>0.999
Any adverse respiratory events^o^	16	15 (34.1)	14	9 (10.0)	Relative risk = 0.29 (0.14 to 0.62)	**0.001**
Severity of TEAE^p^					—	**<0.001**
Grade 1	30	13 (29.5)	37	22 (24.4)	—	**—**
Grade 2	23	20 (45.5)	11	9 (10)	—	—
≥Grade 3	2	2 (4.5)	0	0 (0)	—	—
Severity of TEAE	—	2 [1, 2]	—	0 [0, 1]	Median difference = −1 (−1 to −1)	**<0.001**
TRAE	51	30 (68.2)	38	25 (27.8)	Relative risk = 0.41 (0.28 to 0.60)	**<0.001**
Severity of TRAE^p^					—	**<0.001**
Grade 1	30	12 (35.3)	32	20 (22.2)	—	**—**
Grade 2	19	16 (47.1)	6	5 (5.6)	—	—
≥Grade 3	2	2 (5.9)	0	0 (0)	—	—
Severity of TRAE	—	1 [1, 2]	—	0 [0, 1]	Median difference = −1 (−1 to −1)	**<0.001**
Injection pain	—	27 (61.4)	—	6 (6.7)	Relative risk = 0.11 (0.05 to 0.24)	**<0.001**
Severity of injection pain^q^	—		—		—	**<0.001**
0	—	17 (38.6)	—	84 (93.3)	—	—
1	—	14 (31.8)	—	6 (6.7)	—	—
2	—	11 (25.0)	—	0 (0)	—	—
3	—	2 (4.5)	—	0 (0)	—	—
Severity of injection pain	—	1 [0, 2]	—	0 [0, 0]	Median difference = −1 (−1 to −1)	**<0.001**

Data are median [interquartile range] or *n* (%). TEAE, treatment emergent adverse event; TRAE, treatment-related adverse event; CI, confidence interval.

a Calculated as the ciprofol group versus or minus the propofol group. *P* values in bold indicate statistical differences between the two groups.

b At least one adverse event happened on one patient.

c Judged by the doctor empirically according to clinical manifestations.

d Defined as respiratory rate <8 counts per minute.

e Defined as SpO_2_ <95% on 3 L/min oxygen.

f Defined as absence of breathing action for more than 10 s.

g Defined as respiratory rate >20 counts per minute.

h Defined as systolic pressure <90 mmHg, diastolic pressure <50 mmHg, or mean arterial pressure decrease of ≥20% from baseline.

i Heart rate <50 beats/min or a decrease of >30% from baseline.

j Heart rate >100 beats/min or an increase of >30% from baseline.

k Judged from 12-lead electrocardiograph.

l Defined as the development of any nausea or retching after surgery.

m Newly developed skin rash after surgery requiring therapeutic intervention.

n Defined as verbal rating scales for postoperative pain ≥3.

o Including respiratory depression, desaturation, apnea, and tachypnea.

p Severity of the most serious adverse events that happened on the same patient.

q Evaluated during administration of the first dose of study drugs with a 4-point scale (0 = no pain; 1 = mild pain; 2 = moderate pain; 3 = severe pain).

## Discussion

Results of this phase III randomized trial showed that, for adult patients undergoing ambulatory gynecological procedures, the efficacy of ciprofol used for induction and maintenance of sedation was not inferior to propofol. Ciprofol sedation required more time to reach adequate depth during induction and to reach full awake and PACU discharge criteria during recovery. However, these prolongations were clinically acceptable. On the other hand, ciprofol sedation was associated with less dissatisfaction of patients, less decrease in MAP, less injection pain, and less adverse events especially respiratory events.

Patients undergoing gynecological/obstetrical procedures usually have a high level of anxiety (18, 19). Our finding that the efficacy of ciprofol sedation was non-inferior to propofol was in line with previous studies. In a trial of 109 patients undergoing intubated general anesthesia, three different doses of ciprofol (0.3, 0.4, and 0.5 mg/kg) were used for anesthesia induction and achieved 100% success rate which was comparable to propofol (2.0 and 2.5 mg/kg) (5). The non-inferiority of ciprofol 0.4 mg/kg to propofol 2.0 mg/kg in anesthesia induction was also confirmed in a multi-center trial including 176 patients for elective surgery (10).

In the present study, the median time to successful induction was 2 s longer with ciprofol. Similar results were reported by others. For example, when compared with propofol 2.0 mg/kg in patients undergoing colonoscopy, the mean time to colonoscope insertion was longer with ciprofol 0.4 mg/kg (1.9 min vs. 1.5 min, *p* < 0.01) but was similar with a higher dose ciprofol 0.5 mg/kg (1.5 min) (6). Using midazolam (0.03 mg/kg) and sufentanil (0.3 μg/kg) ahead of ciprofol 0.4 mg/kg or propofol 2 mg/kg resulted in shortened and comparable induction time between the two groups (34.8 ± 15.5 s vs. 35.4 ± 9.5 s, *p* = 0.83) (11). So, with ciprofol, time required to achieve successful induction could be reduced by increasing drug dose or adding other sedatives and analgesics.

Of our patients, median time intervals to full awake and PACU discharge were both 2.3 min longer in the ciprofol group. A previous trial including 267 patients who underwent fiberoptic bronchoscopy also found that time required to full alertness (median 8.50 min vs. 6.00 min, *p* = 0.012) and discharge (median 13.00 min vs. 9.87 min, *p* = 0.002) were longer in patients given ciprofol 0.4 mg/kg than in those given propofol 2.0 mg/kg (9). The recovery time of ciprofol anesthesia is dose-dependent, that is a higher dose requires a longer time of recovery (4). The situation is especially true in patients with renal failure such as those undergoing kidney transplantation (16). However, when anesthesia maintenance was guided by BIS monitoring, recovery time was comparable between ciprofol and propofol anesthesia (13).

Patients receiving propofol for procedure sedation are at risk of adverse events due to limited circumstance and staff (20, 21). When ciprofol 0.4 mg/kg was used in gastroscopy and colonoscopy, the reported incidences of adverse events ranged from 31.3 to 48.4% (6, 8). In our results, both the incidence and severity of either treatment-emergent or treatment-related adverse events were lower in the ciprofol group than those in the propofol group. Importantly, patients receiving ciprofol sedation were less likely to develop desaturation and had higher MAP during the procedures. This indicated that ciprofol dose adopted in our trial was relatively safe considering its dose-dependent respiratory and circulated depression effects (12, 13, 22).

According to previous studies, 37 to 82.3% of patients experienced injection pain during propofol administration (23–25). Intravenous lidocaine could alleviate propofol-related injection pain (24), but was associated with metallic taste which negatively affected patients’ satisfaction (26). Injection pain of propofol was determined by many factors, including the site of injection, size of vein, speed of injection, propofol concentration in the aqueous phase, and the buffering effect of blood (27). As an oil-in-water emulsion, ciprofol has a high hydrophobicity and a low blood plasma concentration, which might explain the reduction of injection pain (1, 2). In accord with previous studies (3, 22, 28), we also found a much lower incidence of injection pain during the induction phase. Low rate of patient dissatisfaction with ciprofol might also be attributed to less injection pain during induction since 52 to 90.9% of patients could recall injection pain after awakening (29–31).

According to our results and previous studies, ciprofol may be a suitable alternative of propofol when used for procedure sedation in patients who are at risk of circulatory instability or respiratory suppression, and those who are sensitive to injection pain. There are some limitations in the present trial. One is that only fentanyl was used in combination with the study drugs. The mutual interaction of ciprofol with other drugs cannot be inferred from this trial. In consideration of patients’ safety, anesthesiologists who were responsible for study drug administration were not blind to treatment assignments. So, the determination to provide supplemental dose might be influenced by personal experience, causing bias to the results. We only enrolled patients with few comorbidities. This limited the generalizability of our results, for example, to aged or critically ill patients.

## Conclusion

Ciprofol was not inferior to propofol when used for sedation in adult patients undergoing ambulatory gynecological procedures. Ciprofol sedation required longer time during induction and recovery, although clinically acceptable, but produced less injection pain and adverse events.

## Data availability statement

The raw data supporting the conclusions of this article will be made available by the authors, without undue reservation.

## Ethics statement

The studies involving humans were approved by Biomedical Research Ethics Committee of Peking University First Hospital and other participating centers. The studies were conducted in accordance with the local legislation and institutional requirements. The participants provided their written informed consent to participate in this study.

## Author contributions

JX: Formal analysis, Investigation, Writing – original draft, Writing – review & editing. MY: Investigation, Writing – original draft. YZ: Investigation, Writing – original draft. X-HZ: Investigation, Writing – original draft. J-HR: Investigation, Writing – original draft. ZX: Investigation, Writing – original draft. H-HX: Investigation, Writing – original draft. Y-HY: Investigation, Writing – original draft. M-JX: Investigation, Writing – original draft. WC: Resources, Writing – original draft. D-XW: Methodology, Supervision, Writing – review & editing.

## Footnotes

^1^https://www.nmpa.gov.cn/datasearch/home-index.html#category=yp

## Supplementary material

SUPPLEMENTARY FIGURE S1 Variations of MAP during sedation and recovery period.

SUPPLEMENTARY FIGURE S2 Variations of HR during sedation and recovery period.

SUPPLEMENTARY FIGURE S3 Variations of SpO_2_ during sedation and recovery period.
