# Supplementary figures and images for: Efficacy and safety of ciprofol for sedation in outpatient gynecological procedures: a phase III multicenter randomized trial
Source: Front Med (Lausanne). 2024 Apr 23;11:1360508. doi: 10.3389/fmed.2024.1360508 (PMC11075489; doi:10.3389/fmed.2024.1360508)

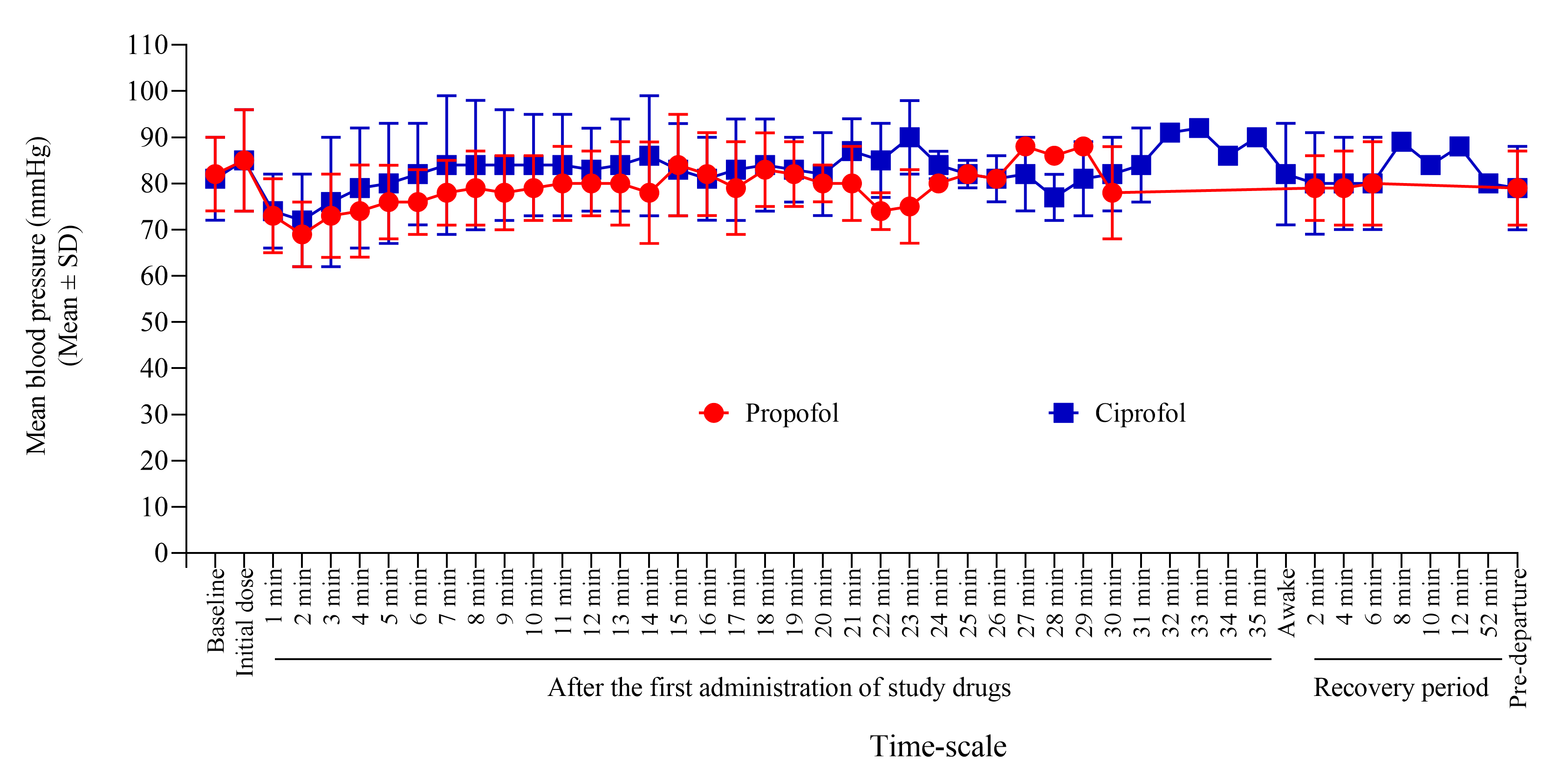

Supplement: SUPPLEMENTARY FIGURE S1 — Variations of MAP during sedation and recovery period. [file Image_1.tif]

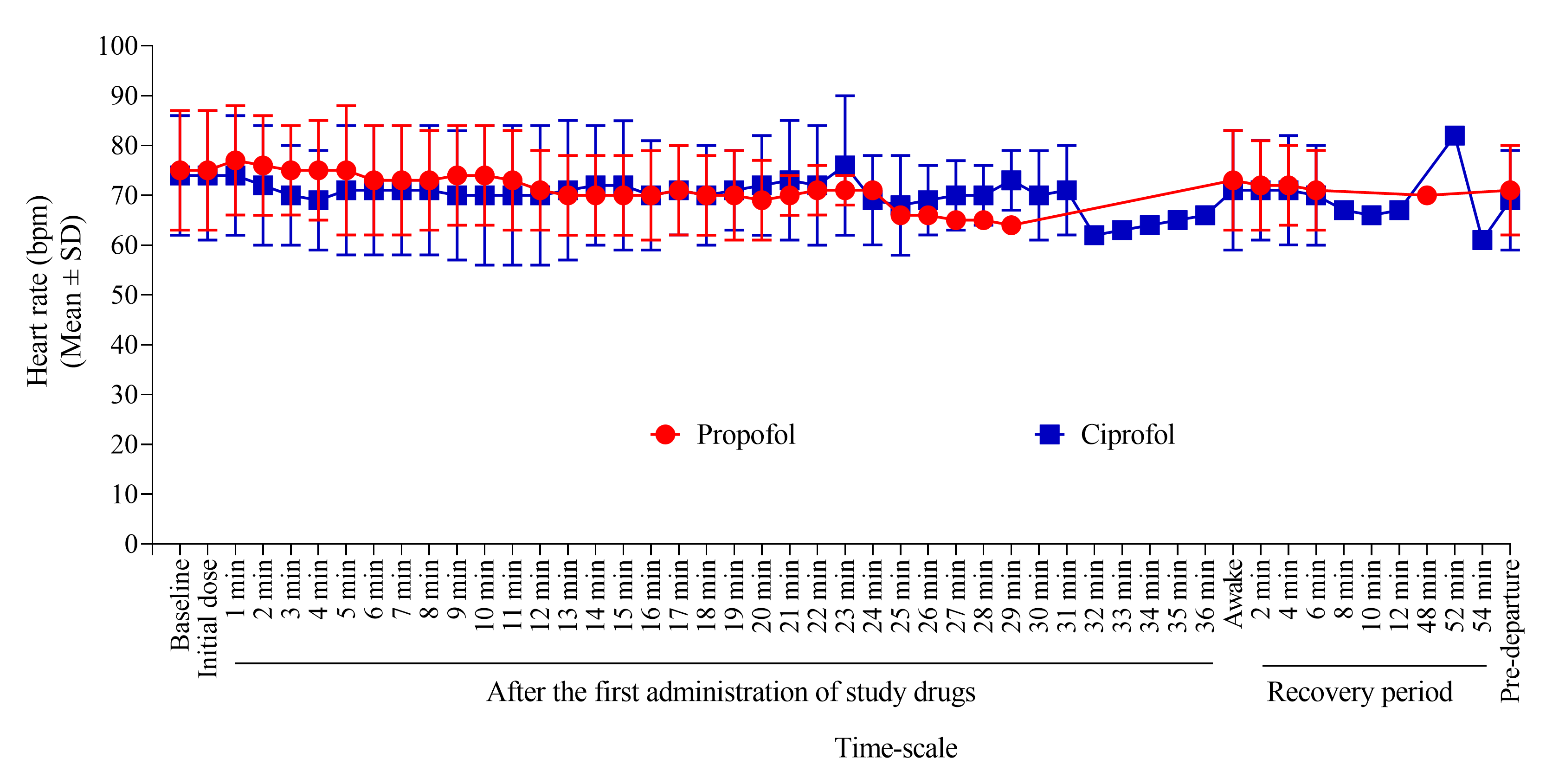

Supplement: SUPPLEMENTARY FIGURE S2 — Variations of HR during sedation and recovery period. [file Image_2.tif]

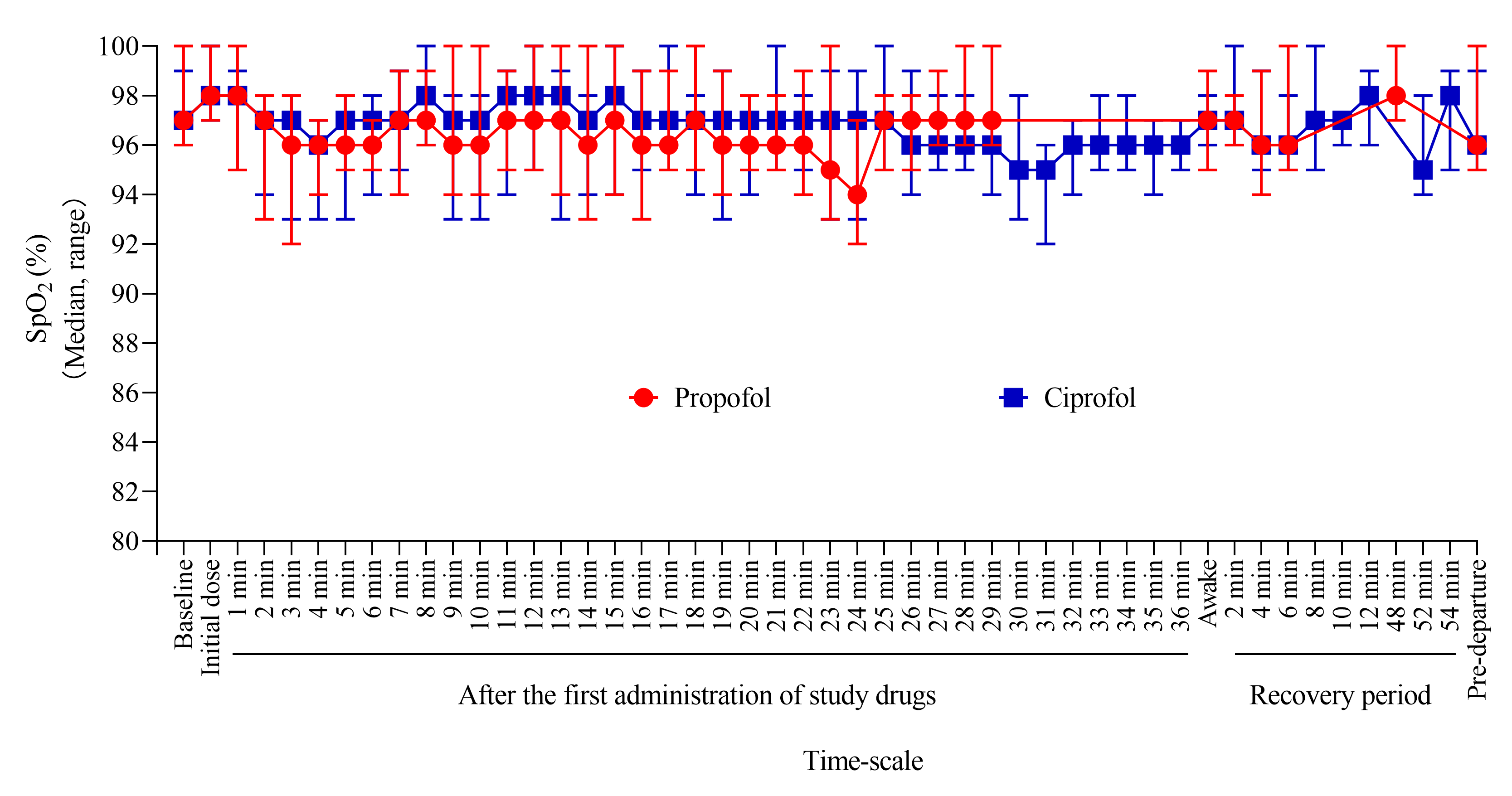

Supplement: SUPPLEMENTARY FIGURE S3 — Variations of SpO2 during sedation and recovery period. [file Image_3.tif]
