# Supplementary material for: Efficacy and safety of ciprofol for sedation in outpatient gynecological procedures: a phase III multicenter randomized trial
Source: Front Med (Lausanne). 2024 Apr 23;11:1360508. doi: 10.3389/fmed.2024.1360508 (PMC11075489; doi:10.3389/fmed.2024.1360508)
Supplement: Supplementary file 4 [file Data_Sheet_1.DOCX]

**Supplementary File 1.** Inclusion and exclusion criteria.

1. **Inclusion criteria**

*Patients who met all the following criteria were considered for inclusion:*

1. Female aged ≥ 18 and ≤ 65 years.
2. Body mass index (BMI) ≥ 18 and ≤ 30 kg/m^2^.
3. Vital signs during the screening period met the following criteria:
   1. Respiratory rate ≥ 10 and ≤ 24 breaths/min.
   2. Blood oxygen saturation (SpO_2_) ≥ 95% when inhaling room air.
   3. Systolic blood pressure (SBP) ≥ 85 mmHg and ≤ 140 mmHg.
   4. Diastolic blood pressure (DBP) ≥ 50 mmHg and ≤ 90 mmHg.
   5. Heart rate ≥ 50 beats/min and ≤ 100 beats/min.
4. American Society of Anesthesiologists (ASA) Classification Ⅰ or Ⅱ.
5. Outpatients requiring gynecological surgery under non-intubated general anesthesia.
6. Patients who understood the procedures and methods of the trial, were willing to complete the trial in strict accordance with the protocol, and signed the written informed consents.
7. **Exclusion criteria**

*Patients who met any of the following criteria during the screening period were excluded:*

1. Any contraindication to general anesthesia or a previous history of anesthesia accidents.
2. Known sensitivity to propofol injection, ciprofol injection, excipients in the investigational drugs (soybean oil, glycerin, triglyceride, egg lecithin, purified lecithin, sodium oleate, sodium hydroxide and disodium edetate), and opioids or any ingredient of opioids; contraindications to propofol.
3. Positive results of urine HCG or blood HCG tests (except outpatients scheduled for surgery to terminate a pregnancy such as curettage, uterine clearance, etc.).
4. Any of the following conditions, which may increase sedation/anesthesia risk:
   1. History of cardiovascular diseases: uncontrolled hypertension (SBP > 140 mmHg and/or DBP > 90 mmHg despite antihypertensive treatment), severe arrhythmia, heart failure, Adams-Stokes syndrome, unstable angina, myocardial infarction within 6 months, previous tachycardia/bradycardia requiring medication, third-degree atrioventricular block, or QTcF ≥ 450 ms (corrected using Fridericia's formula) during the screening period.
   2. History of respiratory disorders: respiratory insufficiency, bronchospasm requiring treatment within 3 months, acute respiratory tract infection with obvious symptoms such as fever, wheezing or productive cough within 1 week.
   3. History of neurological and psychiatric disorders: craniocerebral injury, convulsions, epilepsy, intracranial hypertension, cerebral aneurysm, or cerebrovascular accidents; schizophrenia, mania, chronic use of antipsychotics, or cognitive impairment.
   4. History of gastrointestinal diseases: gastrointestinal retention, active hemorrhage, or other conditions that may lead to reflux and aspiration.
   5. History of uncontrolled and clinically significant liver, kidney, blood, metabolic, or nervous system diseases that were judged to be unsuitable for study participation by the investigators.
   6. History of alcohol abuse within 3 months. Alcohol abuse was defined as daily drinking > 2 units of alcohol (1 unit = 360 mL of beer or 45 mL of liquor with 40% alcohol or 150 mL of wine).
   7. History of drug abuse within 3 months.
   8. Severe infection, trauma, or underwent major surgery within 4 weeks.
5. Any one of the following respiratory risk factors:
   1. History of asthma or stridor.
   2. Sleep apnea syndrome.
   3. History or family history of malignant hyperthermia.
   4. History of failed tracheal intubation.
   5. Estimated difficult airway or difficult tracheal intubation (modified Mallampati score of Ⅲ or Ⅳ).
6. Receiving any of the following medications or therapies:
   1. Participated in other clinical drug trials within 1 month.
   2. Received propofol, other sedatives/anesthetics, opioids, or compounds containing opioid analgesics within 3 days.
7. Laboratory test results that met any of the following criteria:
   1. Neutrophil count < 1.5 × 10^9^/L.
   2. Platelet count < 80 × 10^9^/L.
   3. Hemoglobin < 90 g/L (no blood transfusion within 14 days).
   4. Alanine aminotransferase (ALT) and aspartate aminotransferase (AST) > 3.0 × upper limit of normal (ULN).
   5. Total bilirubin > 2 × ULN.
   6. Serum creatinine > 1.5 × ULN.
8. Women who were pregnant or breastfeeding; women or men of child-bearing potential who were unwilling to use contraception during the trial; subjects or their partners who were planning a pregnancy within 1 month after completion of the trial.
9. Subjects with any other conditions who were judged to be unsuitable for trial participating by the investigators.
