# Supplementary material for: Efficacy and safety of ciprofol for sedation in outpatient gynecological procedures: a phase III multicenter randomized trial
Source: Front Med (Lausanne). 2024 Apr 23;11:1360508. doi: 10.3389/fmed.2024.1360508 (PMC11075489; doi:10.3389/fmed.2024.1360508)
Supplement: Supplementary file 5 [file Table_1.DOCX]

**Supplementary Table 1.** Modified Observer’s Assessment of Alert/Sedation (MOAA/S).

| **Description** | **Score** |
| --- | --- |
| Responds readily to name spoken in normal tone | 5 (alertness) |
| Lethargic response to name spoken in normal tone | 4 |
| Responds only after name is called loudly or repeatedly | 3 |
| Responds only after mild prodding or shaking | 2 |
| Responds only after squeezing the trapezius | 1 |
| Does not respond after squeezing the trapezius | 0 |

**Supplementary Table 2.** Satisfaction evaluation scale by patients.

| **Item** | **Description** | **Score** |
| --- | --- | --- |
| Do you remember when the operation started? | Yes | 0 |
|  | No | 1 |
| Were you conscious during the operation? | All the time | 0 |
|  | Occasionally | 1 |
|  | Never | 2 |
| Did you feel discomfort or pain during the procedure? | No | 3 |
|  | Mild | 2 |
|  | Moderate | 1 |
|  | Severe | 0 |
| Do you remember the end of the operation? | Yes | 0 |
|  | No | 1 |
| If you have minor outpatient surgery in the future, would you be willing to use this anesthetic again? | Yes | 1 |
|  | No | 0 |
| How do you feel about anesthesia/sedation during your treatment? | Satisfied | 2 |
|  | Fair | 1 |
|  | Not satisfied | 0 |

**Supplementary Table 3.** Satisfaction evaluation scale by anesthesiologists.

| **Item** | **Description** | **Score** |
| --- | --- | --- |
| Do you think the subjects were sufficiently anesthetized during the operation? | Too deep/light | 0 |
|  | Fair | 1 |
|  | Enough | 2 |
| Were you satisfied with the duration and procedure of anesthesia induction? | Satisfied | 2 |
|  | Fair | 1 |
|  | Not satisfied | 0 |
| Do you think the subjects experienced any discomfort during the anesthesia induction period? | No | 3 |
|  | Mild | 2 |
|  | Moderate | 1 |
|  | Severe | 0 |
| Were you satisfied with the number of top-up doses of the study drugs during the induction period? | Satisfied | 2 |
|  | Fair | 1 |
|  | Not satisfied | 0 |
| Would you like to use this study drug again in the future when subjects are scheduled for gynecological outpatient surgery? | Yes | 1 |
|  | No | 0 |
| How do you feel about the anesthetics/sedatives employed during the procedure? | Excellent | 2 |
|  | Fair | 1 |
|  | poor | 0 |

**Supplementary Table 4.** Satisfaction evaluation scale by surgeons.

| **Item** | **Description** | **Score** |
| --- | --- | --- |
| Do you think the subjects were sufficiently anesthetized during the operation? | Too deep/light | 0 |
|  | Fair | 1 |
|  | Enough | 2 |
| Do you have any difficulties during the operation on the subject? | Yes, severe | 0 |
|  | Yes, fair | 1 |
|  | No | 2 |
| Do you think the subjects experienced any discomfort during the procedure? | No | 3 |
|  | Mild | 2 |
|  | Moderate | 1 |
|  | Severe | 0 |
| Do you think the subjects tolerate the surgery? | Tolerable | 2 |
|  | Fair | 1 |
|  | Intolerable | 0 |
| Would you like to use this study drug again in the future when subjects are scheduled for gynecological outpatient surgery? | Yes | 1 |
|  | No | 0 |
| How do you feel about the anesthetics/sedatives employed during the procedure? | Excellent | 2 |
|  | Fair | 1 |
|  | poor | 0 |
